# Heterotopic Mineralization of the Medial Collateral Ligament: Our Experience Treating Two Cases of Calcific Versus Ossific Lesions With Ultrasonic Vacuum Debridement

**DOI:** 10.7759/cureus.36127

**Published:** 2023-03-14

**Authors:** Kyungje Sung, Altamash E Raja, Justin G Tunis, Brandon G Tunis, Kevin Zheng, Walter I Sussman

**Affiliations:** 1 Orthopaedics and Rehabilitation Medicine, Tufts Medical Center, Boston, USA; 2 Rehabilitation Medicine, Rowan-Virtua School of Osteopathic Medicine, Sewell, USA; 3 Orthopedics and Sports Medicine, Geisinger Health System, Scranton, USA; 4 Physical Therapy, Geisinger Orthopedics and Sports Medicine, Scranton, USA; 5 Sports Medicine, Boston Sports & Biologics, Wellesley, USA

**Keywords:** tenex, ultrasonic percutaneous debridement, hydroxyapatite calcification, pellegrini-steida lesion, chronic mcl sprain

## Abstract

Chronic injury to the medial collateral ligament (MCL) is common following an acute knee injury. This case report presents two patients that failed to respond to conservative treatment with clinical evidence of an MCL injury and radiographic finding of a benign-appearing soft tissue lesion in the MCL. Calcified or ossified lesions have been described with chronic MCL injuries. Ossification and calcification of the MCL have been observed as potential causes of chronic MCL pain. Here, we detail the distinction between these two distinct intra-ligamentous heterotopic deposits and describe a novel treatment approach using ultrasonic percutaneous debridement, a technique that is typically reserved for tendinopathies. In both cases, pain improved, and they were able to return to their prior level of function.

## Introduction

The medial collateral ligament (MCL) is commonly injured in the knee, with ligamentous injuries accounting for about 40% of knee injuries [1]. Specifically, a 10-year study found that MCL lesions make up 7.9% of all sports-related knee injuries [2]. Most MCL injuries improve with conservative management [3], however, chronic MCL pain has been described in up to 20% of patients following an acute injury [4]. In this report, we present two patients with chronic medial knee pain after an MCL injury, both with intra-ligamentous heterotopic mineralization of the MCL that did not respond to conservative management. Ossification and calcification of the MCL have been described as potential causes of chronic MCL pain, but a clear distinction between the two phenomena is usually not made. Here we compare the ultrasound appearances of these two distinct intra-ligamentous deposits and describe a novel treatment approach.

## Case presentation

Patient 1

A 73-year-old male was referred by an orthopedist for focal, left medial knee pain 12 months after missing a step while descending stairs. He reported a 3/10, intermittent, and sharp pain which worsened with bowling as well as during the first few steps in the morning. Alleviating factors included rest and Ibuprofen. Prior treatments included oral corticosteroids, prescription non-steroidal anti-inflammatory drugs (NSAIDs), and a home exercise program which provided minimal relief. Intra-articular corticosteroid injection was unhelpful. He denied associated swelling, locking, buckling, or instability.

**Figure 1:**
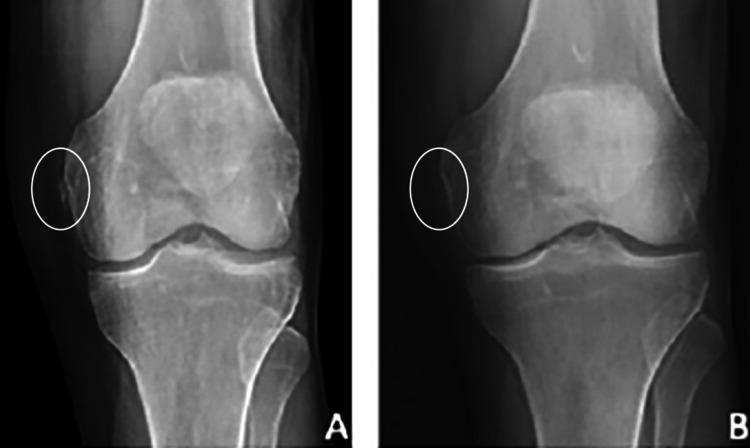
Radiograph Pre-procedure (A) and 6-month post-procedure (B) weight-bearing PA left knee radiograph

Physical examination demonstrated a small joint effusion and medial joint line tenderness with normal range of motion testing, strength testing, and an unremarkable neurovascular examination. Valgus stress yielded no laxity but did reproduce medial knee pain. Patellar grind and passive knee flexion also caused pain, however, the remainder of provocative testing was negative. Left knee radiograph showed a small opacity adjacent to the medial femoral epicondyle but were otherwise unremarkable (Figure 1A). Figure 1 shows pre- and post treatment radiograph images.

**Figure 2:**
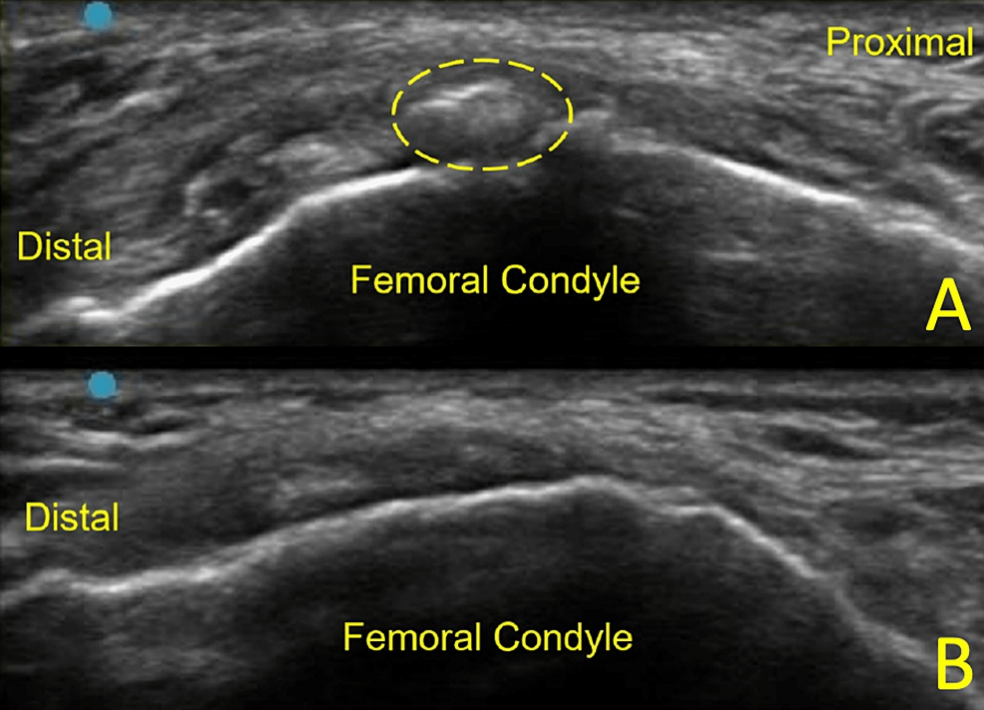
Ultrasound images Patient 1 initial (A), and 6-month post-procedure (B) ultrasound images, long-axis to the medial collateral ligament (MCL) and the calcification (circle)

Diagnostic ultrasound revealed an intra-ligamentous calcification within the proximal MCL without significant thickening of the ligament (Figure 2A). Figure 2 shows pre- and post treatment ultrasound images.

After a lengthy discussion regarding treatment options, the patient decided to proceed with an ultrasound-guided ultrasonic percutaneous debridement. At 10 days post procedure, the patient reported 60 % improvement. At the six-week follow up, the patient stated 90% improvement with only mild discomfort on awakening that subsided after a few steps. He denied any instability, swelling, or limitations with daily activities. By the 6th week, the patient had resumed bowling. After 6 months, the patient reported 99% improvement with occasional discomfort when sleeping in the lateral recumbent position. Repeat radiograph (Figure 1B) and ultrasound imaging (Figure 2B) at this time demonstrated near-complete resolution of the calcification. He endorsed complete satisfaction with the procedure, as it allowed him to return to his previous level of function and perform optimally in his weekly bowling league.

Patient 2

A 46-year-old male was injured as he was getting out of water and was hit by a rogue wave causing valgus stress on the right knee. He was initially seen in an urgent care facility and placed in a knee immobilizer. Ten days later, he presented to the sports medicine clinic with medial knee pain and tenderness over the medial femoral condyle, but no associated extra-articular swelling. There was no joint effusion. Range of motion, strength, and neurovascular examination were unremarkable. There was no laxity with valgus stress, and provocative testing was negative. Initial X-rays of the knee were unremarkable.

**Figure 3:**
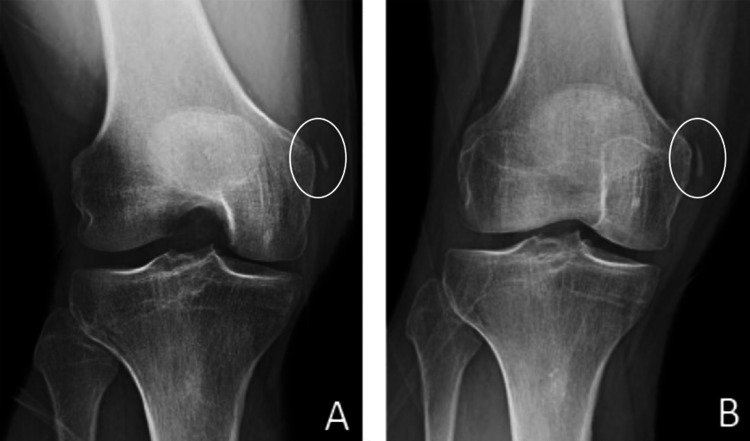
Radiograph Pre-procedure (A) and post-procedure (B) weight bearing PA radiograph with the knee in 45 degrees of flexion. Based on Mendes et al. classification, likely a type I PS lesion

Despite a period of rest and non-steroidal anti-inflammatory drugs (NSAIDs) the patient continued to have intermittent sharp pain over the medial knee, especially when rising from a chair. Magnetic resonance imaging (MRI) performed one month from the date of injury showed patellofemoral chondromalacia and a grade 2 sprain of the MCL with most of the fibers intact. Physical therapy was prescribed, but at five months, the patient continued to have pain. An intra-articular cortisone injection provided no relief and repeat knee radiographs demonstrated a Pellegrini-Stieda (PS) lesion (Figure 3A).

**Figure 4:**
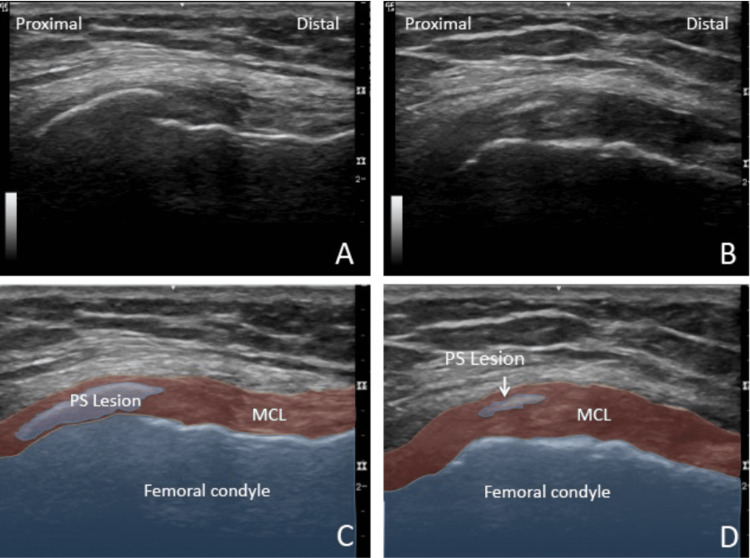
Ultrasound images Long-axis to the medial collateral ligament (MCL) showing the Pellegrini-Stieda (PS) lesion within the ligament

Diagnostic ultrasound showed thickening of the proximal MCL and confirmed an intra-ligamentous ossification within the proximal MCL (Figure 4).

Treatment options were discussed, including continued conservative management, ultrasonic percutaneous debridement, or open surgical debridement. The patient decided to proceed with the ultrasound-guided ultrasonic percutaneous debridement approximately six months from the initial injury.

Two weeks post-procedure, the incision healed completely, and the patient reported 80% improvement in pain. At the 8-week follow-up, the patient reported 90% improvement and X-rays showed an increase in the size and density of the PS lesion compared to pre-procedure X-rays (Figure 3B). At 6- and 12-month follow-ups, the patient reported complete pain relief.

Description of the procedure

The procedure was completed under sterile conditions in an outpatient office for patient 1 and in an ambulatory surgical center for patient 2. Both patients were positioned in the lateral decubitus position, and the intra-ligamentous calcification/ossification was localized with ultrasound (Sonosite Edge II, HFL50x Linear Array Transducer, 6-15 MHz for patient 1 and GE Logiq E, 12L-RS Linear Array Transducer, 5-13 MHz for patient 2). For patient 1, ropivacaine 0.5% (6 mL) was used and for patient 2, a solution of lidocaine 1% without epinephrine (2 mL) and ropivacaine 0.5% (2 mL) was injected around the PS lesion using a 25-gauge 1.5” needle for anesthesia. A stab incision was made with an 11-blade scalpel for the TENEX probe (Tenex Health, Lake Forest, California). In both cases, the probe was introduced through the incision and guided to the pathologic tissue.

**Figure 5:**
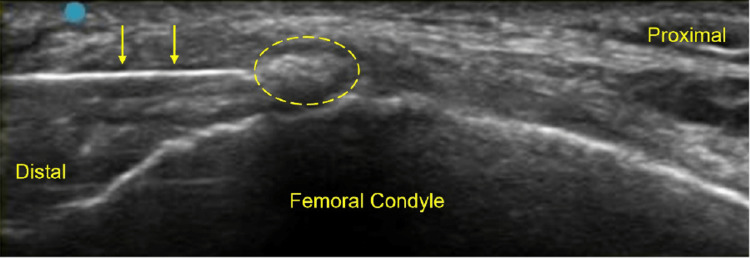
Ultrasound image Demonstrating ultrasonic percutaneous debridement for patient 1. Needle (yellow arrows) directed to the calcification (circle).

**Figure 6:**
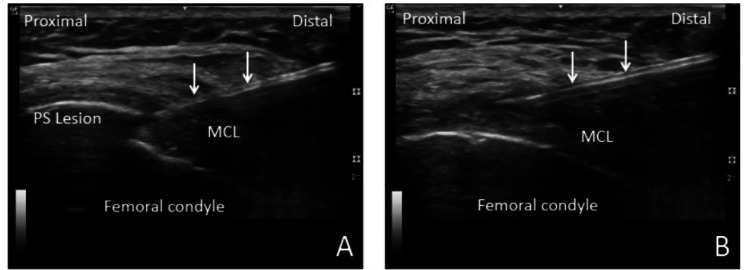
Ultrasound images Demonstrating ultrasonic percutaneous debridement for patient 2. The needle (arrows) was directed to the Pellegrini-Stieda (PS) lesion (A) and the hypoechoic thickened degenerative medial collateral ligament (MCL) (B).

The TENEX probe was activated by depressing the foot pedal, and the diseased tendon was fragmented and removed with a total energy time of 2:05 minutes and 1:38 minutes for patients 1 (Figure 5) and 2 (Figure 6), respectively. The stab incision was closed with a Steri-Strip (3M, St. Paul, Minnesota) followed by gauze and Tegaderm for both patients. The patients were allowed to return to normal activity as tolerated without restrictions. No bracing or formal physical therapy was prescribed. The patients were instructed to discontinue NSAIDs one week prior and six weeks post-procedure. Post-procedure pain was managed with cryotherapy, acetaminophen, and tramadol.

## Discussion

MCL sprains are classified based on instability (Table 1), and chronic MCL pain that lacks instability presents a therapeutic dilemma. Incomplete tears (grades I and II) and isolated tears (grade III) without instability are typically treated non-operatively [5,6], and surgical management is typically reserved for patients with instability [5,7]. In both cases presented here, the patients had an orthopedic surgery consultation for chronic medial knee pain and were not surgical candidates due to the lack of instability. Both patients were found to have heterotopic mineralization of the MCL.

**Table 1:** MCL Classification Systems Two different classifications of medial collateral ligament (MCL) sprains have been described [8,9]

Fetto and Marshall: MCL sprains are classified based on laxity with valgus stress and the knee in 0° and 30° of flexion		
Grade I	No valgus laxity in 0° and 30° of knee flexion	
Grade II	Valgus laxity in 30° of knee flexion, but stable in 0°	
Grade III	Valgus laxity at in 0° and 30° of knee flexion	
Hughston: MCL sprains are classified based on severity (Grade I, II, and III) and laxity (Grade 1+, 2+, and 3+)		
Grade I	First-degree tear, involving a few fibers with localized tenderness, but stable	
Grade II	Second-degree tear, involving more fibers with generalized tenderness, but stable	
Grade III	Third-degree tear, complete ligament rupture with instability Grade III injuries are further subdivided by laxity with the amount of joint separation from valgus stress in 30° of knee flexion	Grade 1+: 3-5 mm of absolute medial separation
		Grade 2+: 6-10 mm of absolute medial separation
		Grade 3+: >10 mm of absolute medial separation

Heterotopic mineralization of the MCL can be due to either calcification (patient 1) or ossification (patient 2) of the ligament, and pose a diagnostic challenge [10,11]. Calcified or ossified lesions in the MCL are not frequently reported in the literature and can be radiographically indistinguishable. These deposits can have varied appearances and locations, but pathologically, calcifications involve the deposition of calcium salts and ossification suggest osseous bone formation [12].

Historically, mineralization of the MCL on radiographs has been described as Pellegrini-Stieda lesions, named after early twentieth century Italian and German surgeons that described ‘ossifications’ of the MCL at or near its proximal insertion of the medial femoral condyle [13], and are characterized based on location and shape (Table 2) [14]. PS lesions have also been described in the tendon of the adductor magnus muscle, medial gastrocnemius muscle or medial patellofemoral ligament [15], and are often associated with trauma [16]. Refractory cases of PS lesions may require surgical debridement of the lesion. However, the surgical literature on the management of PS lesions is limited, with most cases reported before 1965 [15]. Challenges to surgical approaches include high rates of recurrence [17,18], and larger lesions may require surgical reconstruction of the MCL [17]. Delaying surgery and allowing the lesion to mature (~ 12 months) before removing the ectopic bone may decrease the risk of recurrence [19].

**Table 2:** Radiographic descricription of Pellegrini-Steida Lesions MCL: Medial collateral ligament

PS Lesion:	Appearance:	Location:
Type 1	“beak-like”	Extends into the MCL
Type 2	“tear-drop”	Localizes within the MCL, but detached from the femoral condyle
Type 3	“elongated”	Above femur within the adductor magnus tendon
Type 4	“beak-like”	Extends into the MCL and adductor magnus tendon

Hydroxyapatite calcifications of the MCL are uncommon, and to the authors' knowledge, only 11 case reports have described calcification of the MCL [16,20-29]. Calcifications are thought to be caused by soft tissue hypoxia leading to fibrocartilaginous metaplasia, cellular necrosis, and deposition of hydroxyapatite crystals [20]. Corticosteroid injections and shockwave therapy have been described as treatment modalities [21]. Radiographically, hydroxyapatite calcifications can be indistinguishable from detached PS lesions (type II, III or IV lesions), but hydroxyapatite calcifications are a distinct clinical entity and should be differentiated from PS lesions as their pathophysiology likely differs [22,23]. Soft tissue calcifications and ossifications are usually detected with plane films but have a similar radiopaque appearance. MRIs can help rule out associated pathology but can overlook small lesions.

In our two cases, diagnostic ultrasound provided additional clinical information. PS lesions generally have posterior acoustic shadowing, whereas calcifications may have a faint, partial, or absent acoustic posterior shadowing [21,30], and findings can vary depending on whether the calcification is in the inactive (i.e. precalcific or calcific) phase or the resorptive phase [16]. In the inactive phase, calcifications are dense and have more acoustic shadowing, which can limit the clinician’s ability to differentiate between an ossification and calcification. In these cases where the distinction is unclear, the term “heterotopic mineralization” should be used to encompass both ossified lesions and calcifications of the MCL.

There are limited treatment options described for the management of chronic MCL pain, and refractory pain may require surgical management. Open excision of PS ossific lesions can be challenging [17], and there is limited data for managing refractory MCL calcifications with arthroscopic resection or ultrasound-guided percutaneous lavage [20,23,25-27]. Percutaneous treatment of calcific tendinopathy is not a new concept with the procedure being performed with fluoroscopic guidance since the 1970s [31] and with ultrasound guidance since the 1990s [32]. In rotator cuff calcifications, ultrasound-guided percutaneous fenestration [33], lavage or needle barbotage [34], and ultrasonic barbotage have been described [35,36]. Ultrasonic percutaneous debridement is not a novel technology, and in one study, the ultrasonic approach required fewer repeat procedures or subsequent surgery compared to the traditional barbotage group for rotator cuff calcific tendinopathy [36]. In this retrospective study, the authors used the TENEX microtip to emulsify the pathologic tissue and to both irrigate and aspirate the pathologic tissue. The authors also found that the percutaneous ultrasonic barbotage procedure was a quicker and more efficacious in removing the calcifications with the ultrasonic microtip than traditional barbotage [36].

To the authors' knowledge, percutaneous ultrasonic barbotage has not been described for MCL calcification. In this case report, patient 1 had resolution of the calcification within the MCL after the percutaneous ultrasonic debridement, but patient 2 had persistence of the heterotopic mineralization despite the ultrasonic debridement. The lesion in patient 2 was hard, and the physician could not advance the microtip into the lesions, which was consistent with a heterotopic ossification or a PS lesion. The size and density of the heterotopic mineralization did not significantly change after the procedure (Figure 3). Previous studies of PS lesions have reported that in addition to ossification at the enthesis, there are degenerative changes to the ligament [19]. In addition to treating chronic tendinopathy [37-39], percutaneous ultrasonic debridement has been described for the management of chronic MCL sprains [40]. The authors suspect that the clinical improvement in these cases was also due to healing of the pathologic chronic MCL pain after the ultrasonic debridement. The use of needling to treat tendinosis has been well documented. The theory behind the procedure is that repetitively passing the needle through the area of pathology disrupts the chronic degenerative process, causes bleeding and inflammation, and increases growth factors; thus, converting the chronic degenerative process into an acute condition, making it more likely to heal [41]. Fenestration and needling have not been well studied on ligaments, but in one study, a combination of corticosteroid injection and fenestration of incomplete MCL injuries has been shown to result in 96% of patients having an immediate and sustained return to sports [42]. The authors suspect that the clinical improvement in this case was also due to healing of the pathologic chronic MCL pain after the ultrasonic debridement.

## Conclusions

Chronic MCL pain has limited treatment options. The authors believe these cases highlight important potential imaging features and the main consideration of ossified or calcified lesions in chronic MCL pain. In both cases, the patients had a significant and sustained improvement in pain after the ultrasonic debridement procedure. The results of these cases suggest that we should approach these distinct pathologic processes with different treatment goals. In cases of MCL ossification, the outcome may not require the removal of bony pathology and should focus on the debridement of the pathologic tissue within ligament. In contrast, with MCL calcification, the objective should be to remove the calcification. Further studies are needed to validate these treatment modalities for chronic MCL pain, calcifications and ossifications. 
